# Neuroinflammation and synapse loss determines the cognitive fate in Alzheimer Disease

**DOI:** 10.1002/alz.089168

**Published:** 2025-01-03

**Authors:** Sunny Kumar, Ibai Diez, Ana Claudia Amaral, Cinthya Aguero, Charles Zachary Klein, Margaret Scapellato, Riddhimaa Sinha, Teresa Gomez‐Isla

**Affiliations:** ^1^ Massachusetts General Hospital, Boston, MA USA; ^2^ Mass General Institute for Neurodegenerative Disease, Charlestown, MA USA; ^3^ Harvard Medical School, Boston, MA USA; ^4^ Gordon Center for Medical Imaging, Massachusetts General Hospital, Boston, MA USA; ^5^ Massachusetts General Hospital, Harvard Medical School, Boston, MA USA

## Abstract

**Background:**

Some individuals can tolerate the presence of Alzheimer disease neuropathologic changes (ADNC) (e.g., plaques and tangles) without developing dementia. A better understanding of the mechanisms underlying this brain resilience to ADNC may inform the development of novel disease biomarkers and therapies. We investigated the differences in brain changes and gene expression profiles associated with opposite cognitive outcomes (dementia vs. no dementia) in the presence of ADNC.

**Methods:**

We studied postmortem brain samples from 103 participants in the Religious Orders Study (ROS) and Rush Memory and Aging Project (MAP). They all had undergone longitudinal antemortem cognitive assessments. At autopsy, 51 fulfilled neuropathologic criteria for high likelihood of AD (25 demented and 26 cognitively normal ‘resilient’), and 52 fulfilled criteria for low likelihood of AD (26 demented ‘frail’ and 26 cognitively normal ‘controls’). Cases were matched for age, gender, level of education, and co‐pathologies (vascular lesions, Lewy bodies, and TDP‐43 aggregates). Detailed quantitative neuropathological and biochemical assessments and bulk RNA‐seq‐based transcriptomic analyses were conducted in superior temporal and dorsolateral prefrontal cortices.

**Results:**

Demented AD had a similar number of tau tangles compared to resilient but a significantly higher tau neuropil thread burden and soluble hyperphosphorylated tau oligomer levels in synapse‐enriched fractions. The burden of CD68+ microglia was significantly higher in demented AD and frail but not in resilient compared with control brains. Demented AD and frail had a higher loss of synapses than resilient compared with control brains. These differential brain changes paralleled a significant reduction in resilient compared to demented AD in the expression of genes associated with apoptotic, neuroinflammation/response to cytokines, and protein phosphorylation processes. Levels of soluble hyperphosphorylated tau oligomers in synapses and CD68+ microglia burden emerged as the strongest correlates with synaptic densities and antemortem measures of cognition.

**Conclusions:**

These findings suggest that enhanced tau pathology in neurites and synapses may instigate glial inflammatory responses and synapse elimination, determining the cognitive fate of individuals who harbor ADNC.

